# Mesoamerican nephropathy as a potential contributor to chronic allograft dysfunction in tropical settings

**DOI:** 10.3389/ti.2026.16621

**Published:** 2026-04-29

**Authors:** Arriel Makembi Bunkete

**Affiliations:** 1 Centre Hospitalier Universitaire de Guyane, Site de Saint-Laurent-du-Maroni, Saint-Laurent-Du-Maroni, French Guiana; 2 Department of Nephrology, University Clinics of Kinshasa, University of Kinshasa, Kinshasa, Democratic Republic of Congo; 3 Departement of Home Hemodialysis, Renal Care Unit (RCU), Saint-Laurent-Du-Maroni, French Guiana

**Keywords:** chronic allograft dysfunction, chronic kidney disease (CKD), kidney transplantation (KT), mesoamerican nephropathy, tropical nephropathy

Dear Editors,

Chronic kidney disease (CKD) represents a major and growing global health burden, affecting well over 850 million individuals worldwide and contributing substantially to morbidity and mortality [1, 2]. Despite advances in diagnostic approaches, a significant proportion of end-stage renal disease (ESRD) cases remains classified as of unknown etiology, particularly in low-resource and tropical settings. This diagnostic uncertainty is especially problematic in the context of kidney transplantation, where unidentified causal factors may persist after transplantation and compromise long-term graft survival.

The under-recognition of environmental and occupational nephropathies may exacerbate this issue in tropical regions like French Guiana. Among these, Mesoamerican nephropathy (MeN) emerges as a plausible but largely overlooked contributor. Initially described in Central American agricultural workers, MeN predominantly affects young individuals without diabetes or hypertension and is characterized by chronic tubulointerstitial injury, low-grade proteinuria, and progressive decline in renal function [3]. Its pathogenesis is complex and multifactorial, involving repeated exposure to heat stress, chronic dehydration, environmental nephrotoxins such as pesticides and heavy metals, and recurrent subclinical acute kidney injury [4, 5] (Table 1).

**TABLE 1:** Pathophysiological mechanisms of Mesoamerican nephropathy and potential impact on kidney allograft function.

Mechanism	Renal effects	Potential impact on kidney allograft
Recurrent dehydration and renal hypoperfusion	Tubular ischemia, reduced GFR	Repeated subclinical injury leading to chronic graft dysfunction
Recurrent subclinical acute kidney injury	Progressive tubulointerstitial fibrosis	Accelerated decline in graft function
Heat stress	Tubular inflammation, cellular stress	Increased susceptibility of graft to environmental injury
Environmental nephrotoxins (pesticides, heavy metals)	Direct tubular toxicity	Synergistic damage with immunosuppressive nephrotoxicity
Oxidative stress	Cellular damage, fibrogenesis	Promotion of chronic allograft fibrosis
Chronic inflammation	Interstitial remodeling and fibrosis	Long-term structural deterioration of graft
Hyperosmolarity and electrolyte disturbances	Tubular stress and injury	Functional instability of the graft

Although environmental determinants are central to current models, Mesoamerican nephropathy is increasingly recognized as a multifactorial condition in which individual susceptibility may also play a role. Emerging evidence suggests that genetic and epigenetic factors may modulate vulnerability to renal injury and influence disease progression [6]. Candidate genes involved in endothelial function and vascular regulation, such as *APOE* and *NOS3*, have been proposed as potential contributors to interindividual variability in susceptibility. In addition, epigenetic mechanisms, including DNA methylation changes—particularly in regions of chromosome 7—may influence gene expression in response to chronic environmental stressors such as heat exposure and dehydration [6].

The environmental and occupational conditions observed in French Guiana—including high ambient temperatures, persistent humidity, and physically demanding labor in agricultural, construction, or forest-related activities—closely resemble those described in endemic regions of MeN. These similarities strongly support the hypothesis that a comparable entity, sometimes referred to as “Meso-Amazonian nephropathy,” may be present but underdiagnosed in this setting. Epidemiological data indicate that a non-negligible proportion of ESRD cases in French Guiana remain of indeterminate origin, raising the possibility that unrecognized MeN contributes to this burden [7].

This hypothesis has important implications for kidney transplantation. When the etiology of native kidney disease is unknown, transplant recipients may remain exposed to the same pathogenic factors that led to initial renal failure. In the case of MeN, continued exposure to heat stress, inadequate hydration, and physically demanding occupational conditions may directly affect the transplanted kidney. Such exposure may result in repeated episodes of subclinical renal hypoperfusion and injury, ultimately leading to progressive functional decline despite appropriate immunosuppressive therapy and standard clinical follow-up.

In this context, genetic susceptibility may also influence transplant outcomes. Variability in donor and recipient genetic profiles has been shown to affect graft survival through mechanisms related to immune response, vascular integrity, and susceptibility to injury [8]. While polygenic risk scores are currently primarily used for highly heritable renal diseases, their potential extension to multifactorial conditions such as MeN could provide a more refined framework for risk stratification. In particular, incorporating genetic susceptibility into donor evaluation may, in the future, contribute to improved donor selection and better anticipation of graft vulnerability. Although the implementation of such approaches remains challenging in resource-limited tropical settings due to economic and infrastructural constraints, they may represent a valuable long-term strategy for optimizing transplant outcomes in high-risk populations, particularly by enhancing the understanding of genetic factors that influence transplant success.

The present letter highlights MeN as a potential non-immunological and underappreciated determinant of chronic allograft dysfunction in tropical settings. Unlike traditional causes of graft failure, such as acute or chronic rejection, immunological incompatibility, or drug-related nephrotoxicity, this mechanism is primarily environmental and therefore theoretically preventable. However, it remains largely absent from current transplant evaluation frameworks and follow-up strategies, which rarely incorporate detailed environmental or occupational risk assessments, potentially leading to missed opportunities for improving graft outcomes in tropical settings, such as the failure to identify and mitigate specific environmental factors that could contribute to chronic allograft dysfunction (Figure 1).

**FIGURE 1:**
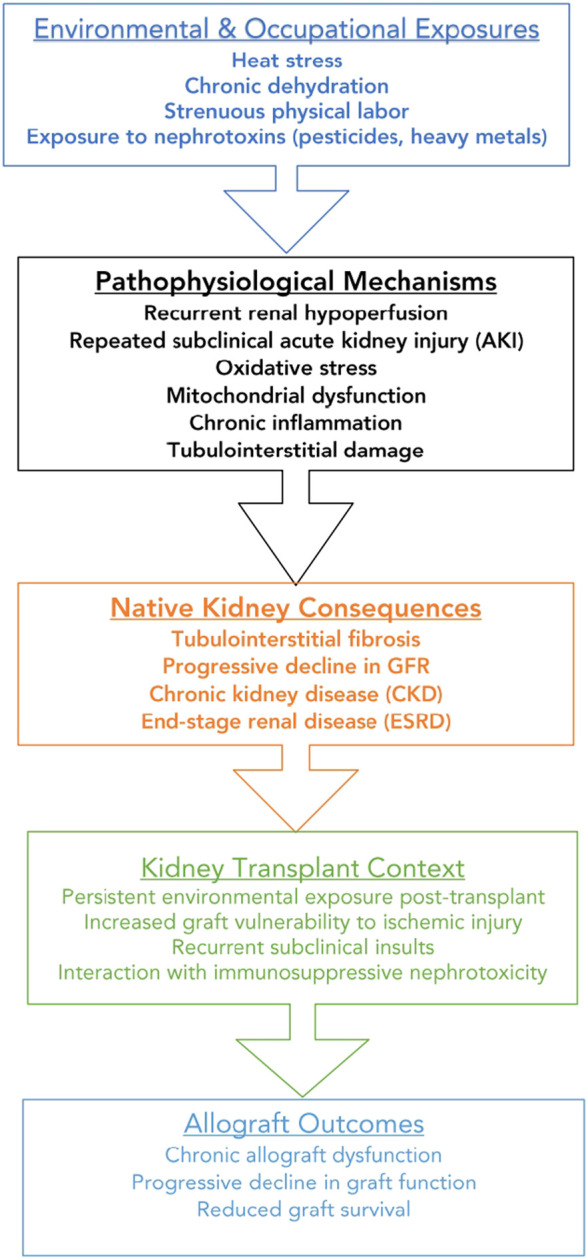
Conceptual framework of environmental and pathophysiological mechanisms linking Mesoamerican nephropathy to native and kidney allograft dysfunction.

In French Guiana and similar tropical territories, this issue is further compounded by a range of contextual challenges. Transplant recipients are exposed to a high burden of infectious diseases, including tuberculosis, HIV, and endemic parasitic infections, which may reactivate or worsen under immunosuppressive therapy [9]. In addition, healthcare delivery is often constrained by geographical remoteness, limited access to specialized care, and reduced availability of diagnostic tools, which may delay the detection and management of graft dysfunction [9]. Socioeconomic factors also play a critical role, as many patients face financial pressures that necessitate early return to physically demanding work after transplantation, thereby increasing exposure to heat stress and dehydration [10]. These factors may interact synergistically with MeN-related mechanisms to accelerate graft injury and functional decline, leading to poorer long-term outcomes for transplant patients, particularly in those who are already vulnerable due to their socioeconomic status and limited access to healthcare resources.

Importantly, the relevance of Mesoamerican nephropathy may extend beyond tropical regions to the European context. Increasing migration from Central and South America, as well as from other tropical regions exposed to extreme occupational heat stress, may result in more kidney transplant candidates or recipients with unrecognized environmentally mediated kidney injury in European healthcare systems. In parallel, climate change is contributing to more frequent and intense heat waves across Europe, particularly in Southern regions, potentially recreating environmental conditions similar to those implicated in Mesoamerican nephropathy. These converging phenomena underscore the need for transplant programs in Europe to incorporate environmental and occupational exposure histories into pre- and post-transplant risk assessment strategies.

Recognizing MeN as a potential contributor to graft outcomes calls for a more comprehensive and context-adapted approach to transplant care. Pre-transplant evaluation should include a detailed assessment of occupational history, environmental exposures, and lifestyle factors, particularly in patients with ESRD of unknown etiology. Identifying individuals at risk could allow for targeted counseling and implementation of preventive strategies. Post-transplant management should emphasize strict hydration, avoidance of prolonged heat exposure, and adaptation of occupational activities whenever feasible. Patient education is essential, as awareness of environmental risk factors remains limited in many affected populations, which can lead to increased complications and poorer health outcomes if not addressed through effective communication and resources.

From a pathophysiological standpoint, the mechanisms underlying MeN are relevant to transplanted kidneys, which may be particularly vulnerable to repeated subclinical insults. These mechanisms include recurrent renal hypoperfusion due to dehydration, cumulative tubular injury, oxidative stress, mitochondrial dysfunction, and progressive interstitial fibrosis [4]. A concise summary of these mechanisms and their potential impact on graft function is provided below.

In addition to conventional clinical approaches, emerging technologies such as digital health tools and artificial intelligence may offer new opportunities to improve risk stratification and follow-up in resource-limited settings. By integrating environmental, occupational, and biological data, predictive models could help identify patients at increased risk of graft dysfunction and enable earlier and more personalized interventions [11]. Remote monitoring strategies may also help overcome geographical barriers and improve continuity of care.

Considering MeN in the evaluation of ESRD etiology and in post-transplant follow-up represents an important step toward more personalized and context-specific care in tropical environments. Beyond its clinical implications, this perspective emphasizes the necessity to broaden current paradigms of graft dysfunction by incorporating environmental determinants, such as local disease prevalence and socio-economic factors, into routine practice. Additional epidemiological, clinical, and translational studies are required to validate the existence and assess the impact of MeN in French Guiana and analogous areas, as well as to formulate targeted preventive and therapeutic strategies [12].

Raising awareness of this potential entity among transplant clinicians and integrating environmental risk assessment into clinical practice may ultimately contribute to improved graft survival and better long-term outcomes for patients living in tropical settings.

## Data Availability

The original contributions presented in the study are included in the article/supplementary material, further inquiries can be directed to the corresponding author.
